# Protective association of the *HIF-1α* rs11549465 polymorphism with metabolic syndrome in people living with HIV on antiretroviral therapy

**DOI:** 10.3389/fmed.2026.1802450

**Published:** 2026-03-31

**Authors:** Nemanja Đorđević, Jovan Ranin, Ivana Gmizić, Marko Marković, Ivan Rajković, Jovana Ranin, Biljana Ljujić, Marina Kostić, Ivana Lešnjak, Nataša Minić, Biljana Popovska Jovičić

**Affiliations:** 1 Department of Infectious Diseases, Faculty of Medical Sciences, University of Kragujevac, Kragujevac, Serbia; 2 Clinic for Infectious Diseases, University Clinical Centre Kragujevac, Kragujevac, Serbia; 3 Department of Infectious Diseases, Faculty of Medicine, University of Belgrade, Belgrade, Serbia; 4 Clinic for Infectious and Tropical Diseases, University Clinical Centre of Serbia, Belgrade, Serbia; 5 Department of Genetics, Faculty of Medical Sciences, University of Kragujevac, Kragujevac, Serbia; 6 Department of Pharmacology and Toxicology; Centre for Research on Harmful Effects of Biological and Chemical Hazards, Faculty of Medical Sciences, University of Kragujevac, Kragujevac, Serbia; 7 Department of Psychiatry, Faculty of Medical Sciences, University of Kragujevac, Kragujevac, Serbia; 8 Psychiatric Clinic, University Clinical Centre Kragujevac, Kragujevac, Serbia

**Keywords:** antiretroviral therapy, HIF-1α, HIV, hypoxia, hypoxia-inducible factor, metabolic syndrome, rs11549465

## Abstract

**Background:**

People living with HIV (PLWH) receiving antiretroviral therapy (ART) are at increased risk of metabolic syndrome (MS). Hypoxia-inducible factor 1 alpha (HIF-1α) is involved in metabolic regulation, and the functional polymorphism of *HIF-1α* rs11549465 (C1772T) may influence susceptibility to metabolic disturbances. This study aimed to investigate the association between the *HIF-1α* rs11549465 polymorphism and MS in PLWH on ART.

**Methods:**

This multicentre cross-sectional study included 121 PLWH. Participants were classified as MS cases or controls according to NCEP ATP III criteria. Genotyping of the *HIF-1α* rs11549465 polymorphism was performed using Real-time PCR with a TaqMan assay. Clinical, metabolic, and HIV-related parameters were analysed. Multivariable logistic regression was used to identify independent factors associated with MS.

**Results:**

MS was identified in 47 participants (38.8%), while 74 (61.2%) served as controls. The minor T allele frequency of *HIF-1α* rs11549465 was 24%. The presence of T allele and CT/CT+TT genotypes was associated with a significantly lower odds of MS (*p* < 0.05). Among the participants with MS, carriers of CT+TT exhibited a lower prevalence of hypertension (*p* < 0.05). The individuals with MS were older and had longer HIV infection duration and ART exposure (*p* < 0.05). Stratified analysis indicated that the protective association of the CT genotype was more pronounced at shorter durations of HIV infection and shorter ART exposure (*p* < 0.05). In multivariable analysis, elevated triglycerides and fasting glycemia were the strongest independent correlates of MS, while the *HIF-1α* rs11549465 CT+TT genotype remained independently associated with lower odds of MS (*p* < 0.05).

**Conclusion:**

The *HIF-1α* rs11549465 T allele and CT+TT genotypes are associated with a lower odds of prevalent MS and hypertension in PLWH receiving ART. This protective association remained evident after adjustment for established metabolic risk factors, although it appeared less pronounced with longer HIV infection and ART duration.

## 1 Introduction

Metabolic syndrome (MS) is a constellation of interrelated biochemical and clinical abnormalities, including central adiposity, insulin resistance (IR), dyslipidemia, elevated blood pressure, and proinflammatory and prothrombotic state. Collectively, these alterations are associated with a twofold increase of coronary artery disease risk and a fivefold increase of diabetes mellitus (DM) risk (1, 2). In the general population, MS represents a growing global health burden, with an estimated prevalence ranging from 11.2% to 45.4% worldwide (3) and approximately 24.3% in European populations (4). In Central Serbia, the reported prevalence in the general population is around 25%; however, comprehensive national data remain limited (5).

People living with HIV (PLWH) are disproportionately affected by MS with a 1.6-fold higher risk compared with the general population (6). This risk is particularly pronounced among individuals receiving antiretroviral therapy (ART), who exhibit 1.5-fold higher odds compared with untreated PLWH (6). Beyond traditional metabolic risk factors, chronic immune activation and persistent inflammation are increasingly recognized as key contributors to metabolic dysfunction in this population.

Excessive abdominal and perivisceral fat accumulation, a core component of MS, leads to adipose tissue hypoxia (ATH), adipocyte dysfunction, and dysregulated adipocytokine secretion, ultimately promoting IR and systemic inflammation (1, 7). Hypoxia-inducible factor 1 alpha (HIF-1α) is a heterodimeric transcription factor composed of an oxygen-sensitive alpha (α) subunit and a constitutively expressed beta (β) subunit. The *HIF-1α* gene, located on chromosome 14, encodes the HIF-1α protein that plays a central role in cellular adaptation to hypoxia by regulating genes involved in glucose metabolism, angiogenesis, inflammation, and mitochondrial function (8). Under conditions of ATH, HIF-1α is stabilized and its intracellular activity is increased, particularly within expanded adipose tissue (8). This activation promotes IR, chronic low-grade inflammation, and metabolic dysregulation, thereby providing a mechanistic link between hypoxia and the development of MS (8).

However, data regarding the role of HIF-1α in obesity remain conflicting. While some studies suggest that HIF activation exacerbates IR and inflammation, others indicate that stabilizing HIF may improve metabolic outcomes. These discrepancies likely arise from differences in experimental models, tissue-specific effects, and the complex, context-dependent nature of HIF signaling. In DM, tissues are exposed to hypoxic conditions: however, HIF-1 signaling is often dysregulated, resulting in impaired adaptive responses. Hyperglycemia and elevated free fatty acid levels can inhibit both the stability and transcriptional activity of HIF-1α, although in certain cell types HIF-1α may also be activated through a carbohydrate response element-binding protein-dependent mechanisms (9).

Chronic inflammation and immune activation remain key characteristics of HIV infection, despite effective viral suppression with modern ART. Previous studies have demonstrated that cytosolic viral double-stranded (dsDNA) generated during HIV replication in infected CD4-positive (CD4+) T lymphocytes induces mitochondrial reactive oxygen species (ROS) production, leading to increased HIF-1α activity. This cascade promotes enhanced viral replication and triggers the release of extracellular vesicles that activate bystander CD4 + T lymphocytes to secrete IFN-*γ* and stimulate macrophages to produce pro-inflammatory cytokines, including interleukin-6 (IL-6) and interleukin-1β (IL-1β), thereby creating a chronic inflammatory milieu even in individuals receiving ART with undetectable viral load (10). In addition, the HIV viral proteins such as Tat (in infected neurons) and gp120 and Vpr (in glial cells) can induce ROS production and consequently activate HIF-1α (11–13).

Alongside metabolic and inflammatory pathways, genetic variability has been thoroughly investigated in HIV research. Polymorphisms in host genes, such as CCR5, as well as sequence variability in viral regulatory proteins including Tat and Vif, have been shown to influence HIV pathogenesis and host–virus interactions (14–17). While these studies examine different clinical outcomes, they demonstrate the broader influence of genetic variation on HIV-related phenotypes.

Furthermore, single-nucleotide polymorphisms (SNPs) in the *HIF-1α* gene can influence HIF-1α expression and function. The rs11549465 polymorphism (C1772T, Pro582Ser) represents a missense variant, in which the minor T allele exhibits increased transactivation activity under both normoxic and hypoxic conditions (18). It has consistently been shown that this polymorphism is both functional and biologically relevant (19–21).

Given the complexity of this issue and the conflicting evidence regarding the role of *HIF-1α* SNPs and HIF-1α protein in the development of MS in the general population, especially in Caucasians, as well as having in mind the lack of data on this association in PLWH, we aimed to investigate the effect of the functional *HIF-1α* (rs11549465) polymorphism on the development of MS in PLWH receiving ART.

## 2 Materials and methods

### 2.1 Study population

This multicentre, cross-sectional genetic association study of *HIF-1α* rs11549465 and prevalent MS included 121 outpatient and hospitalized PLWH treated in the Clinic for Infectious Diseases at University Clinical Centre Kragujevac (UKCKg) and Clinic for Infectious and Tropical Diseases, University Clinical Centre of Serbia (UKCS). Participants were enrolled regardless of gender, severity of HIV infection, or type of ART. The study was conducted between April 2023 and May 2024. The primary inclusion criterion was consistent use of ART for at least 18 months (22). Eligible participants were Caucasian adults of Serbian nationality (≥18 years) with confirmed HIV infection diagnosed by reverse transcriptase polymerase chain reaction (RT-PCR) from blood samples, and without a diagnosis of MS at the time of HIV diagnosis and the initiation of ART. Pregnancy and breastfeeding were exclusion criteria.

Written informed consent was obtained from all participants. All procedures were conducted in accordance with the Declaration of Helsinki and Good Clinical Practice guidelines. The study protocol was approved by the local Ethics Committees of UKCKg, Faculty of Medical Sciences, University of Kragujevac (FMNKg), and UKCS, with decision numbers No. 01/22-38, 01-3253, and 307/31, respectively.

### 2.2 Data collection

Anthropometric and clinical measurements, as well as blood sampling for laboratory analyses, were performed during a single routine outpatient examination (index visit). Demographic and HIV-related data were retrospectively extracted from the hospital information system and linked to the index visit. Eligibility required continuous ART use for ≥18 months at study assessment. Participants who had not yet reached this threshold were assessed at a subsequent routine visit once they fulfilled the eligibility criterion. PLWH who did not have an eligible index visit (for example, stopping ART before reaching 18 months) were not included. Demographic and HIV-related data included age, sex, duration of HIV infection, CD4+ lymphocyte count at HIV diagnosis, type and duration of ART, and plasma HIV ribonucleotide acid (RNA) levels measured by quantitative reverse transcription-polymerase chain reaction (RT-PCR). Patients who had an acute infectious disease or infection, as well as an exacerbation of chronic non-infectious diseases or conditions, were not included in the study. Also, critical illness or those who are in the postoperative course are not included. Anthropometric measurements included body weight (BW), body height (BH), and waist circumference (WC), along with laboratory parameters.

The presence of hypertension, dyslipidemia, DM, and current therapy for these conditions was assessed at the index visit. Participants receiving anti-hypertensive, lipid-lowering, or anti-diabetic therapy were classified as hypertensive, dyslipidemic, or hyperglycemic, respectively, regardless of the obtained values in laboratory tests, for grouping purposes and statistical analyses using dichotomous variables. These participants were excluded from analyses based on continuous laboratory measurements. This method was chosen to prevent pharmacologically modified phenotypes that might bias the connection between genotype and untreated metabolic characteristics. BW and BH were measured using a calibrated hospital scale, with barefoot participants wearing light clothing. Body mass index (BMI) was calculated as BW (kg) divided by BH squared (m^2^). WC was measured in centimetres using a non-stretchable tape placed midway between the iliac crest and the lower rib margin at the end of expiration. For descriptive purposes, BMI was categorized as overweight/obesity (BMI ≥ 25 kg/m^2^) (23). This categorization was used for exploratory comparisons and should be distinguished from the central obesity component of MS, defined by WC. Blood pressure (BP) was measured during index visit using an aneroid sphygmomanometer (BP AG1-20, Microlife AG, Swiss Corporation) after at least 15 min of rest.

After a fasting state of at least 12 h, an 8 mL venous blood sample was collected into a serum separation tube (SST) using a Vacutainer system. Serum was used to determine lipid profile parameters, including total cholesterol (TC), low-density lipoprotein cholesterol (LDL-C), high-density lipoprotein cholesterol (HDL-C), triglycerides (TG), and fasting glucose levels. For subgroup analyses, TC and LDL-C were dichotomized based on clinical cut-offs (>5.2 mmol/L for TC and >2.59 mmol/L for LDL-C) to define the presence or absence of elevated cholesterol levels (24). Analyses were performed in the central laboratory of UKCKg using standard methods on a Beckman-Colter AU400 Unicel DXC 800 Synchron Clinical System. An additional 5-ml venous blood sample was collected in an ethylenediaminetetraacetic acid (EDTA) tube for HIV RNA quantification by RT-PCR, used as evidence of sustained adherence to ART. CD4+ lymphocyte counts were determined from EDTA blood samples using a FACS Count system (BD Biosciences, San Jose, CA, USA).

After data collection, the participants were classified according to the National Cholesterol Education Program Adult Treatment Panel III (NCEP ATP III) criteria (25) into an MS group (≥3 criteria fulfilled) or a control group (≤2 criteria fulfilled). Based on the CD4+ T lymphocyte count at HIV diagnosis, the participants were further categorized as non-late presenters (non-LP; CD4+ T Ly ≥ 350 cells/mm^3^), late presenters (LP; CD4+ T Ly 200–349 cells/mm^3^), or individuals with advanced HIV infection/AIDS (<200 cells/mm^3^) in accordance with definitions of the Center for Disease Control and Prevention (CDC) and related criteria (26–29). This classification was used to assess the relationship between immune status, *HIF-1α* polymorphism, and the occurrence of MS.

### 2.3 DNA extraction and SNP genotyping

The *HIF1α SNP* (rs11549465) was selected based on the data from HapMap database,^1^ dbSNP,^2^ and previous studies (30–33). Genetic analyses were performed in a newly established molecular laboratory at UKCKg, adapted for work with infectious material, and at the FMNKg laboratory. Peripheral blood samples were collected in a 5-ml EDTA tubes. Genomic DNA was extracted from 200 μL of EDTA plasma using the GeneJET genomic DNA purification kit (Thermo Scientific), following the manufacturer’s instructions. Genotyping of the *HIF-1α* rs11549465 polymorphism was performed by real-time PCR using a TaqMan SNP Genotyping Assay (C__25473074_10) on an ABI 7500 Real-time System (Applied Biosystems, Foster, California, USA). Manufacturer-validated probes were used. Previously genotyped samples served as positive controls, and No-Template Controls (NTC) were included as a negative controls. Genotype calls were independently assessed by two researchers, with no discrepancies observed.

### 2.4 Statistical analysis

Sample size was calculated using GPower 3.1 software, assuming an effect size of 0.3, a type I error (*α*) of 0.05, and a statistical power of 80% with two degrees of freedom (df) = 2. The calculation was based on a dominant genetic model and incorporated the expected population frequency of the *HIF-1α* rs11549465 CT+TT genotype, accounting for unequal genotype distribution. Due to the lack of comparable published data, estimates were informed by a pilot dataset from the study cohort. Genotype and allele frequencies were expressed as absolute and relative frequencies. Due to a low frequency of the TT genotype, the dominant model (CT+TT vs. CC) was selected in advance as the main in statistical analyses. Other genetic models (allelic, additive, recessive) were analysed for descriptive comprehensiveness and are regarded as exploratory. Deviation from Hardy–Weinberg equilibrium was assessed using an exact test (Guo & Thompson) due to low frequency of the TT genotype. Statistical analyses were performed using SPSS software, version 26 (IBM, Armonk, NY, USA). Categorical variables were analysed using the Chi-squared test or Fisher’s exact test, as appropriate, while continuous were compared using the Mann–Whitney U test. Associations between independent variables and the presence of MS were assessed by calculating odds ratios (OR) with 95% confidence intervals (CI) using logistic regression analysis. In subgroup analyses with smaller sample sizes, ORs and 95% CIs were derived from 2 × 2 contingency tables, and Fisher’s exact test was used to assess statistical significance. Univariable logistic regression was conducted initially, followed by multivariable logistic regression. A primary multivariable logistic regression model was constructed to assess the association between *HIF-1α* rs11549465 and MS, adjusting for demographic and HIV-related confounders. MS-defining components were not included in this model. An additional secondary model incorporating selected metabolic variables was then explored to examine whether the observed association remained evident after accounting for these factors. The model’s assumptions were evaluated by assessing multicollinearity, and goodness-of-fit was examined using the Hosmer–Lemeshow test. Interaction analyses were performed using logistic regression including *HIF-1α* rs11549465 (dominant genetic model), ART class at the index visit (NNRTI as reference; PI and INSTI as dummy variables), and genotype × ART interaction terms. A two-sided *p* < 0.05 was considered statistically significant.

## 3 Results

### 3.1 General characteristics

The median age of the study population was 44 (interquartile range (IQR) 39–54; range 23–78 years). The cohort consisted of 98 males (81%) and 23 females (19%). At the time of the HIV diagnosis, 60 participants (49.6%) were classified as non-LP, while 60 (49.6%) were classified as LP. According to CD4+ T-lymphocyte count and diagnostic criteria, 50 participants (41.3%) were diagnosed at the AIDS stage. The median duration of HIV infection was 10 years (IQR 5–15), and the median duration of ART was also 10 years (IQR 5–15), with a range of 2–31 years for both parameters. At the time of assessment, all participants had undetectable plasma HIV RNA levels. The prevalence of MS in the study was 38.8%. Detailed demographic, clinical, and laboratory characteristics are presented in Table 1.

**Table 1:** Demographic and clinical features.

Variable	Category	Metabolic syndrome *n* = 47 (38.8%)	Controls *n* = 74 (61.2%)	*p*
Demographic data				
Age (years)^a^		51 (44.25–59.5)	41 (37–46)	<0.001^b^
Gender	Males	34 (72.3%)	63 (85.1%)	0.137^c^
	Females	13 (27.1%)	11 (14.9%)	
HIV status				
Non-LP (CD4+ >350 mm^3^)	Yes	22 (46.8%)	38 (51.4%)	0.764^c^
	No	25 (53.2%)	36 (48.6%)	
LP (CD4+ <350 mm^3^)	Yes	25 (53.2%)	35 (47.3%)	0.656^c^
	No	22 (46.8%)	39 (52.7%)	
AIDS (CDC criteria^d^)	Yes	18 (38.3%)	32 (43.2%)	0.727^c^
	No	29 (61.7%)	42 (56.8%)	
HIV infection duration (years)^a^		12.5 (7–16)	9 (5–15)	0.020^b^
ART duration (years)^a^		11.5 (7–16)	8 (5–14)	0.023^b^
Metabolic parameters				
SBP^a,†^ (mmHg)		130 (120–140)	120 (113–130.0)	<0.001^b^
DBP^†^ (mmHg)		80 (80–90)	79 (70–80)	0.001^b^
WC (cm)		101 (95–109)	89 (83–96)	<0.001^b^
BMI^a^ (kg/m^2^)		26.4 (24.5–28.2)	23.7 (21.6–24.9)	<0.001^b^
Laboratory analysis				
TC^a,†^ (mmol/l)		5.48 (4.89–5.93)	4.99 (4.36–5.66)	0.061^b^
LDL- C^a,†^ (mmol/l)		2.96 (2.08–3.64)	2.98 (2.42–3.51)	0.610^b^
HDL- C^a,†^ (mmol/l)		1.17 (1.0–1.4)	1.28 (1.18–1.62)	0.014^b^
TG^a,†^ (mmol/l)		1.90 (1.12–2.47)	1.05 (0.67–1.40)	<0.001^b^
Glycemia^a,†^ (mmol/l)		5.70 (5.05–6.55)	5.20 (5.05–5.55)	<0.001^b^

The results are presented as absolute (*n*) and relative (%) frequency or a median (Md) with interquartile range (IQR). LP, late presenters; CD4^+^, CD4-positive T helper cells; AIDS, acquired immunodeficiency syndrome; CDC, Center for Disease Control and Prevention; ART, antiretroviral therapy; SBP, systolic blood pressure; DBP, diastolic blood pressure; WC, waist circumference; BMI, body mass index; TC, total cholesterol; LDL-C, low-density lipoprotein cholesterol; HDL-C, high-density lipoprotein cholesterol; TG, triglycerides; p, probability value; b, Mann–Whitney U test; c, Pearson Chi-Square; d, according to CDC classification of HIV status; †: PLWH taking antihypertensive, lipid-lowering, and antidiabetic drugs were excluded from analysis. ^a^Denotes variables presented as median (Md) with interquartile range (IQR).

### 3.2 Distribution of alleles and genotypes of the *HIF1α* rs11549465 polymorphism among study groups

In our study population, the allele frequencies of the *HIF1α* rs11549465 polymorphism were 76% for the C allele (184/242) and 24% for the T allele (58/242). The genotype distribution was 54.5% for CC (66/121), 43.0% for CT (52/121), and 2.5% for TT (3/121).

The genotype distribution was consistent with Hardy–Weinberg equilibrium according to the exact test (*p* = 0.077). The distribution of alleles and genotypes according to the presence of MS is presented in Table 2. In univariate logistic regression analysis, the presence of the T allele was associated with reduced odds of MS. Similarly, carriers of the CT genotype and carriers of the combined CT+TT genotypes showed reduced odds of MS compared with CC genotype carriers. At the allele level, the T allele was less frequent among participants with MS compared with controls (17.0% vs. 28.4%). The presence of the T allele was associated with lower odds of MS (OR 0.52, 95% CI 0.27–0.99; *p* = 0.046). Genotype-based analysis using an additive model showed that carriers of the CT genotype had significantly lower odds of MS compared with CC homozygotes (OR 0.39, 95% CI 0.179–0.85; *p* = 0.018). No significant association was observed for the TT genotype, likely due to its low frequency. In the dominant genetic model, carriers of at least one T allele (CT+TT) had significantly lower odds of MS compared with CC homozygotes (OR 0.40, 95% CI 0.19–0.86; *p* = 0.018). In contrast, no significant association was observed under the recessive model.

**Table 2:** Allele and genotype frequencies of the *HIF1α* rs11549465 variant in PLWH.

Genetic model		Allele / Genotype	Metabolic syndrome	Control	OR [95% CI]^†^	*p*	
		Frequency % (n/N)	Frequency % (n/N)			
Alleles					
rs11549465	C	83.0 (78/94)	71.6 (106/148)	1	ref.
	T	17.0 (16/94)	28.4 (42/148)	0.52 [0.27; 0.99]	0.046
Genotypes					
Additive model					
rs11549465	C/C	68.1 (32/47)	45.9 (34/74)	1	ref.
	C/T	29.8 (14/47)	51.4 (38/74)	0.39 [0.18; 0.85]	0.018
	T/T	2.1 (1/47)	2.7 (2/74)	0.53[0.046; 6.15]	0.613
Dominant model					
rs11549465	C/C	68.1 (32/47)	45.9 (34/74)	1	ref.
	C/T+T/T	31.9 (15/47)	54.1 (40/74)	0.40 [0.19; 0.86]	0.018
Recessive model					
rs11549465	C/C+C/T	97.9 (46/47)	97.3 (72/74)	1	ref.
	T/T	2.1 (1/47)	2.7 (2/74)	0.78 [0.07; 8.88]	0.843

The results are displayed as relative frequencies (%) and absolute counts (*n*). The dominant model was predefined as the primary analytical model; other models are shown for completeness. ref, indicates the reference category; OR, Odds ratio; 95% CI, 95% confidence interval. p, denotes probability; †, Univariable logistic regression.

### 3.3 Impact of *HIF1α* rs11549465 polymorphism genotypes on the expression of comorbidities associated with MS

The distribution of MS-associated comorbidities according to *HIF1α* rs11549465 genotype was analysed separately in participants with MS and in controls. Due to the low frequency of the TT genotype, analyses were performed using a dominant genetic model (CC vs. CT+TT). The results are presented in Tables 3, 4. Among the participants with MS, carriers of the CT+TT genotype showed lower odds of hypertension compared with CC genotype carriers (OR 0.30, 95% CI 0.08–1.20; *p* = 0.049). No statistically significant associations were observed between genotype status and other MS-related comorbidities including TC, LDL-C, fasting glycemia, or BMI. Among participants without MS (control group), as it is shown in Table 4, carriers of the CT+TT genotype had higher odds of elevated fasting glycemia compared with CC genotype carriers (OR 4.50, 95% CI 1.10–18.35; *p* = 0.014). No statistically significant associations were observed between genotype status and hypertension, TC, LDL-C, TG levels or BMI.

**Table 3:** Association of *HIF1α* rs11549465 genotypes and MS-related comorbidities in the PLWH with MS.

Comorbidities	Category	CC (n/N, %)	CT+TT (n/N, %)	OR (95%CI) (CT+TT vs. CC) ^†^	*p* ^‡^
Hypertension^§^	Present	16/20 (80.0%)	4/20 (20.0%)	0.30 (0.075–1.20)	0.049
	Absent	12/22 (54.5%)	10/22 (45.5%)		
TC	High	17/24 (70.8%)	7/24 (29.2%)	0.88 (0.24–3.17)	0.832
	Normal	15/22 (68.2%)	7/22 (31.8%)		
LDL - C	High	20/26 (76.9%)	6/26 (23.1%)	0.51 (0.14–1.94)	0.229
	Normal	12/19 (63.2%)	7/19 (36.8%)		
HDL - C^§^	Low	7/10 (70.0%)	3/10 (30.0%)	0.99 (0.19–5.19)	1.0
	Normal	23/33 (69.7%)	10/33 (30.3%)		
TG^§^	High	18/23 (78.3%)	5/23 (21.7%)	0.49 (0.12–2.01)	0.202
	Normal	14/22(63.6%)	8/22 (36.4%)		
Glycemia^§^	Elevated	20/27 (74.1%)	7/27 (25.9%)	0.53 (0.14–2.04)	0.265
	Normal	12/20 (60.0%)	8/20 (40.0%)		
Overweight/obesity (BMI ≥25 kg/m^2^)	Present	23/34 (67.6%)	11/34 (32.4%)	0.84 (0.20–3.55)	1.0
	Absent	7/11 (63.6%)	4/11 (36.4%)		

TC, Total cholesterol; LDL-C, Low-density lipoprotein cholesterol; HDL-C, High-density lipoprotein cholesterol; TG, Triglycerides; BMI, Body Mass Index; OR, Odds ratio; 95% CI, confidence intervals; †, calculation from 2×2 contingency tables; CC. reference category; ‡, Fisher’s exact test; §: defined according to NCEP ATP III criteria.

**Table 4:** Association of *HIF1α* rs11549465 genotypes and MS-related comorbidities in the control group of PLWH.

Comorbidities	Category	CC (n/N, %)	CT+TT (n/N, %)	OR (95% CI) (CT+TT vs CC)^†^	*p* ^‡^
Hypertension^§^	Present	5/7 (71.4%)	2/7 (28.6%)	0.25 (0.04–1.38)	0.102
	Absent	21/55 (38.2%)	34/55 (61.8%)		
TC	High	14/28 (50.0%)	14/28 (50.0%)	1.41 (0.58–3.45)	0.509
	Normal	17/41 (41.5%)	24/41 (58.5%)		
LDL - C	High	22/47 (46.8%)	25/47 (53.2%)	0.85 (0.28–2.60)	0.777
	Normal	9/21 (42.9%)	12/21 (57.1%)		
HDL - C^§^	Low	4/8 (50.0%)	4/8 (50.0%)	1.11 (0.24–5.13)	1.0
	Normal	27/57 (47.4%)	30/57 (52.6%)		
TG^§^	High	3/6 (50.0%)	3/6 (50.0%)	1.21 (0.23–6.48)	1.0
	Normal	29/64 (45.3%)	35/64 (54.7%)		
Glycemia^§^	Elevated	4/19 (21.1%)	15/19 (78.9%)	4.50 (1.10–18.35)	0.014
	Normal	30/55 (54.5%)	25/55 (45.5%)		
Overweight/obesity (BMI ≥25 kg/m^2^)	Present	4/17 (23.5%)	13/17 (76.5%)	2.97 (0.74–11.93)	0.133
	Absent	21/44(47.7%)	23/44 (52.3%)		

TC, Total cholesterol; LDL-C, Low-density lipoprotein cholesterol; HDL-C, High-density lipoprotein cholesterol; TG, Triglycerides; BMI, Body Mass Index; OR, Odds ratio; 95% CI, confidence intervals; †, calculation from 2×2 contingency tables; CC, reference category; ‡, Fisher’s exact test; §, defined according to NCEP ATP III criteria.

### 3.4 Characteristics of applied ART

At the time of ART initiation, 44 of 121 participants (36.4%) were treated with a non-nucleoside reverse transcriptase inhibitor (NNRTI)-based regimen, 47/121 (38.8%) received a protease inhibitor (PI)-based therapy, and 30/121 (24.8%) were treated with integrase strand transfer inhibitors (INSTI)-based regimens. At study entry, after median ART duration of 10 years, 22/121 (18.2%) participants were receiving NNRTI-based regimens, 17/121 (14.0%) PI-based regimens, and 82/121 (67.8%) INSTI-based regimens. During the course of treatment, 59/121 (57.0%) participants had received abacavir-containing therapy at some point. Nucleoside reverse transcriptase inhibitors (NRTIs) were not analysed as a separate category, as they constituted the backbone of nearly all combined ART regimens. In unadjusted analyses, the prevalence of MS differed across ART regimens, with the highest prevalence observed in participants receiving the PI-based regimens (52.9%), followed by those on INSTI-based regimens (40.2%), and the lowest prevalence among those on NNRTI-based regimens (22.7%). However, in unadjusted logistic regression analysis, ART regimen was not significantly associated with MS overall (global *p* = 0.153). Compared with the NNRTI-based regimen as the reference category, higher odds of MS were observed in participants receiving PI-based therapy (OR = 3.83, 95% CI 0.96–15.19; *p* = 0.049), while no statistically significant association was identified for INSTI-based regimens (OR = 2.34, 95% CI 0.79–6.96; *p* = 0.127). An interaction analysis was subsequently performed using logistic regression, including *HIF1α* rs11549465 genotype, ART class (NNRTI/PI/INSTI), and genotype × PI, and genotype × INSTI interaction terms. Neither interaction term reached statistical significance (*p* = 0.105 and *p* = 0.627, respectively). Cross-tabulated genotype × ART cell counts are presented in Supplementary Table 1.

### 3.5 Potential influence of HIV infection duration and ART exposure length on the modulation of the protective effect of the *HIF1α* rs11549465 CT genotype on the development of MS

**Figure 1:**
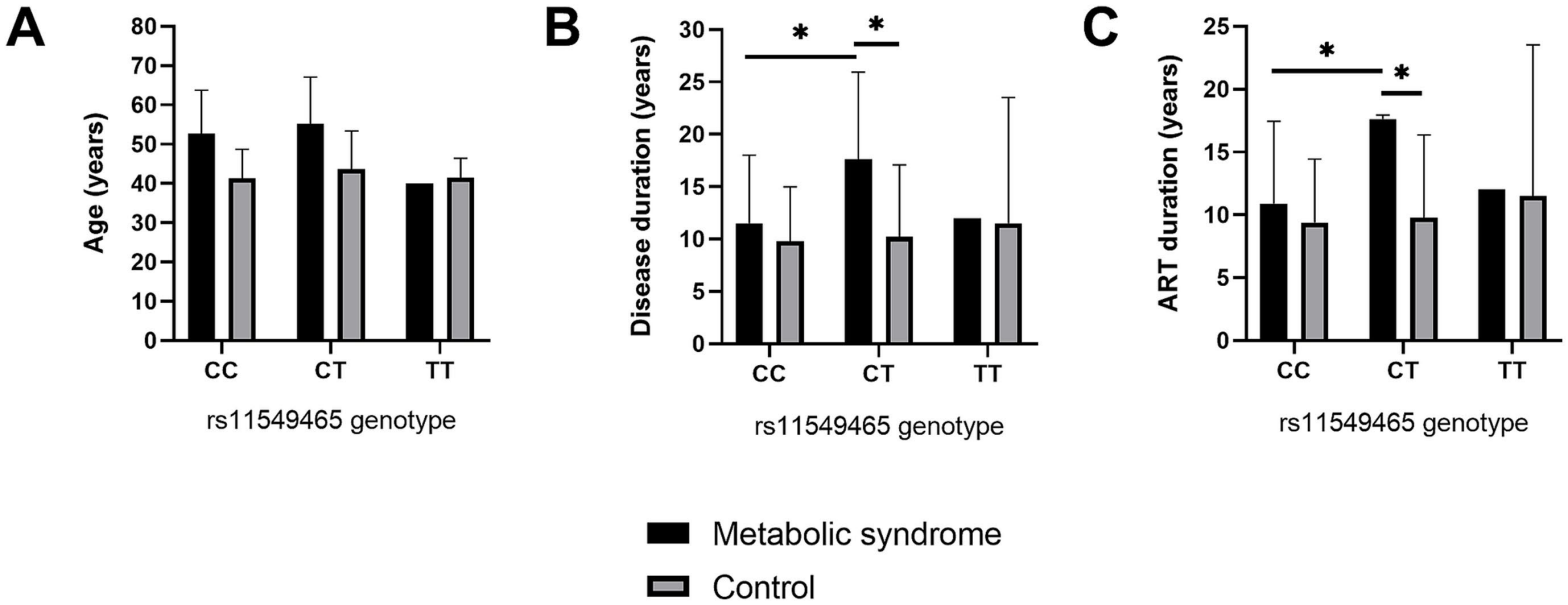
Association of *HIF1α* rs11549465 genotypes with **(A)** age, **(B)** duration of HIV infection, and **(C)** duration of ART exposure in PLWH with and without MS. * denotes statistical significance (*p*<0.05).

As shown in Table 1, the participants with MS were older and had longer duration of HIV infection and longer exposure to ART compared with controls (*p* = <0.001, 0.020, and 0.023, respectively). To assess differences between study groups according to age, duration of HIV infection, and duration of ART exposure across *HIF1α* rs11549465 genotype polymorphism, a two-way analysis of variance (ANOVA) was performed. The results are presented in Figure 1. Among carriers of the CT genotype, participants with MS had a significantly longer duration of HIV infection and longer ART exposure compared with controls (*p* = 0.007, 0.005, respectively). In addition, within the MS group, carriers of the CT genotype had a longer duration of HIV infection and longer exposure to ART compared with CC genotype carriers. No statistically significant differences were observed between MS and control groups among CC or TT genotype carriers for HIV infection duration, ART exposure, or age. Age did not differ significantly between study groups across genotypes.

**Figure 2:**
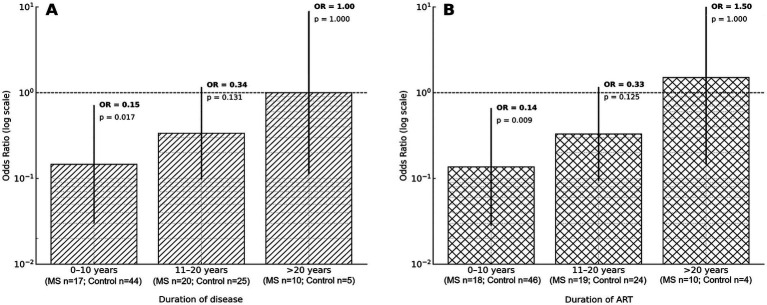
Exploratory stratified analysis of the association between the *HIF1α* rs11549465 CT genotype and prevalent MS according to duration of **(A)** HIV infection and **(B)** ART exposure. Odds ratios (ORs) represent unadjusted estimates comparing CT genotype carriers to non-CT genotypes (reference group) within each duration category. Vertical lines indicate 95% confidence intervals (log scale). Sample sizes for MS and control groups are indicated for each stratum. **(A)** Stratified by duration of HIV infection. In the 0–10-years category, the frequency of the CT genotype was 11.8% (2/17) in MS group and 47.7% (21/44) in the control group (OR = 0.15; 95% CI 0.03–0.72, *p* = 0.017). In the 11–20-years category CT genotype frequency was 30% (6/20) in the MS group and 56% (14/25) in controls (OR = 0.34; 95% CI 0.10–1.16, *p* = 0.131). In the > 20-years category, CT genotype frequencies were 60% (6/10) in the MS group compared to 60% (3/5) in controls (OR = 1.00, 95% CI 0.11–8.95, *p* = 1.000). **(B)** Stratified by duration of ART exposure. In the 0–10-years ART duration category, the frequency of CT genotype was 11.1% (2/18) in MS group and 47.8% (22/46) in the control group (OR = 0.14; 95% CI 0.03–0.66, *p* = 0.009). In the 11–20-years category, CT genotype frequencies were 31.6% (6/19) in the MS group and 58% (14/24) in controls (OR = 0.33, 95% CI 0.09–1.17, *p* = 0.125). In the >20 years ART duration category, CT genotype frequencies were 60% (6/10) in MS compared to 50% (2/4) in controls (OR = 1.50; 95% CI 0.15–15.46, *p* = 1.000).

To further explore the association between the *HIF-1α* rs11549465 CT genotype and MS, a stratified analysis was performed among participants carrying the CT genotype only. The odds of MS were analysed according to the duration of HIV infection and the duration of ART, categorized as ≤10 years, 11–20 years, and >20 years. The results are presented in Figures 2A,B. Among CT genotype carriers with a duration of HIV infection ≤10 years, the odds of MS were significantly lower compared with the reference category (OR 0.15, *p* = 0.017). In the 11-20-years category, the odds of MS were lower but did not reach statistical significance (OR 0.34, *p* = 0.131), while no difference was observed in participants with a duration of HIV infection >20 years (OR 1.00, *p* = 1.000) (Figure 2A). A similar pattern was observed when stratifying by duration of ART exposure. Among CT genotype carriers with ≤10 years of ART exposure, the odds of MS were significantly lower (OR 0.14, *p* = 0.009). In the 11-20-years category, the association was not statistically significant (OR 0.33, *p* = 0.125), and no difference was observed among participants with >20 years of ART exposure (OR 1.50, *p* = 1.000) (Figure 2B).

### 3.6 Overview of variable estimates from the best-fitting multiple logistic regression models addressing MS

A multivariable logistic analysis was performed to determine factors independently associated with the presence of MS. Two regression models were developed with distinct objectives. In the primary model, the *HIF-1α* rs11549465 genotype (dominant model: CT+TT vs. CC) was included as the principal predictor. The model was adjusted for age, sex, initial CD4 + T-cell count, duration of HIV infection and ART exposure, and the ART regimen class on index visit. The results are depicted in Table 5. In this model, the *HIF-1α* rs11549465 CT+TT genotype remained significantly associated with lower odds of MS (OR = 0.32, 95% CI 0.13–0.81, *p* = 0.016). Age was independently associated with MS (OR = 1.11 per year, 95% CI 1.05–1.18, *p* < 0.001), while sex, initial CD4+T-cell count, HIV duration, cumulative ART exposure, and ART class did not show independent associations with MS. The model showed acceptable calibration [Hosmer–Lemeshow *χ*^2^(8) = 12,799; *p* = 0,119], explained a moderate amount of variation in MS status (Nagelkerke *R*^2^ = 34,6%) and correctly classified 71.7% of the participants.

**Table 5:** Primary logistic regression model of factors associated with MS.

Variables	B	S.E.	Wald	*p*	OR	95% CI
rs11549465^†^	−1.132	0.469	5.835	0.016	0.322	0.129–0.808
Age	0.107	0.028	14.717	<0.001	1.112	1.054–1.175
Sex^‡^	0.013	0.589	0.000	0.982	1.013	0.320–3.211
Initial CD4+ T Ly count	0.000	0.001	0.000	0.983	1.000	0.999–1.001
HIV duration	0.003	0.039	0.004	0.947	1.003	0.929–1.082
ART overall			0.262	0.877		
PI-based group^§^	0.428	0.835	0.262	0.609	1.533	0.299–7.875
INSTI-based group^§^	0.220	0.637	0.119	0.730	1.246	0.357–4.346
Consant	−5.276	1.313	16.142	<0.001	0.005	

Ly, lymphocytes; ART, antiretroviral therapy; PI, Protease inhibitors; INSTI, Integrase strand transfer inhibitors; B, the regression coefficient; S.E., the standard error; Wald *χ*^2^, Wald test statistics for the degree of freedom of 1 (df = 1); p, the probability; OR, odds ratio; 95% CI, the 95% confidence interval for the estimated OR; †, dominant genotype, CC, reference category; ‡, male gender as a reference category; §, NNRTI as a reference category.

We further developed a secondary model including *HIF-1α* rs11549465 (CT+TT vs. CC), age, duration of HIV infection, and key metabolic parameters (SBP, BMI, glycemia, and TG) to assess whether the observed association persisted after adjustment for MS-related traits. *HIF-1α* rs11549465 remained significantly associated with MS (OR = 0.18, 95% CI 0.04–0.84, *p* = 0.030). As expected, BMI (OR = 1.56, 95% CI 1.18–2.07, *p* = 0.002), TG (OR = 5.17, 95% CI 1.59–16.82, *p* = 0.006), glycemia (OR = 5.22, 95% CI 1.18–23.07, *p* = 0.029), and SBP (OR = 1.08, 95% CI 1.02–1.14, *p* = 0.013) were independently associated with MS. Age also retained an independent association with MS (OR = 1.15, 95% CI 1.03–1.29, *p* = 0.011), while duration of HIV infection was no longer statistically significant in the presence of the metabolic variables in this model (Table 6). This model demonstrated an adequate fit to the data [Hosmer–Lemeshow test, *χ*^2^(7) = 10.663, *p* = 0.222]. The model explained 78.1% of the variance in MS status (Nagelkerke *R*^2^ = 78.1%) and correctly classified 92.2% of cases.

**Table 6:** Secondary logistic regression model of factors associated with MS.

Variables	B	S.E.	Wald	*p*	OR	95% CI
rs11549465^†^	−1.741	0.801	4.717	0.030	0.175	0.036–0.844
Age	0.142	0.056	6.416	0.011	1.152	1.033–1.286
HIV duration	0.103	0.061	2.850	0.091	1.109	0.984–1.250
SBP	0.075	0.030	6.218	0.013	1.078	1.016–1.143
BMI	0.447	0.143	9.725	0.002	1.563	1.181–2.070
Glycemia^‡^	1.652	0.758	4.742	0.029	5.216	1.180–23.066
Triglycerides	1.643	0.602	7.454	0.006	5.171	1.590–16.819
Constant	−31.760	6.805	21.785	<0.001	0.000	

SBP, Systolic blood pressure; BMI, Body mass index; B, the regression coefficient; S.E., the standard error; Wald *χ*^2^ – Wald test statistics for the degree of freedom of 1 (df = 1); p, the probability; OR, odds ratio; 95% CI, the 95% confidence interval for the estimated OR; †, dominant genotype; CC, reference category; ‡, normal as a reference category, defined by NCEP ATP III criteria.

## 4 Discussion

In this study, the *HIF-1α* rs11549465 polymorphism was independently associated with the presence of MS in PLWH receiving ART. Carriers of the T allele (CT+TT genotypes) exhibited a significantly lower odds of MS, and this association persisted after adjustment for established demographic and clinical confounders in the primary multivariable regression model. Alongside the genetic effect, increasing age and key metabolic parameters—BMI, SBP, fasting glycemia, and TG levels—were independently associated with MS, consistent with its multifactorial pathogenesis. As these results are based on the secondary regression model, they should be interpreted in context, given that some of the included variables overlap with the MS definition. Although the duration of HIV infection showed a tendency toward increased odds of MS, it did not retain statistical significance in our secondary regression model, suggesting that its effect may be mediated through cumulative cardiometabolic factors rather than acting as an independent determinant. Importantly, the protective association of the *HIF-1α* rs11549465 polymorphism persisted even in the presence of strong clinical correlates of MS, supporting a potential modulatory role of hypoxia-related genetic variation in metabolic risk among PLWH.

Advances in early diagnosis and modern ART have enabled durable viral suppression, immune reconstitution, and a substantial extension of life expectancy in PLWH. As a result, this population is increasingly exposed to traditional cardiometabolic risk factors, cumulative ART toxicity, and the long-term effects of chronic HIV infection, all of which contribute to the development of non-communicable comorbidities, such as MS, often at a younger age than in the general population (34). However, the relative contribution of HIV infection itself, ART, and conventional risk factors to MS development remain a matter of debate (35, 36). In our study, participants who developed MS were significantly older than those without MS, supporting previous observations that aging is a major determinant of cardiometabolic risk in PLWH. This finding is consistent with report by Mondy et al. (37), who identified increasing age as the key risk factor for MS in people who live with HIV receiving ART.

In European cohorts of PLWH receiving ART, the prevalence of MS defined according to the NCEP ATP III criteria has been reported to range from approximately 17% in the Catalonian cohort (38) to 33–45% in early HAART-era Italian cohorts, resulting in a pooled regional estimate of about 24% (39, 40). Data on the prevalence of MS among PLWH receiving ART in Serbia remain limited. Existing studies have predominantly focused on individual components of MS rather than on the syndrome as a whole. In this context, Jevtović et al. (41) reported a 29.1% prevalence of lipodystrophy (LD) – an established component of MS – in a large Sebian cohort of PLWH. In our study, the prevalence of MS was 38.8%, exceeding most previously reported estimates in European Caucasian PLWH (42). This difference may reflect variations in cohort characteristics, including age distribution, duration of HIV infection, cumulative exposure to ART, and study period. Although our cohort was predominantly male, no significant gender-related differences were observed, in line with prior findings from predominantly male HIV cohorts (43).

Across Europe, approximately 40%–60% of people diagnosed with HIV present late for care, defined as CD4+ < 350 cells/mm^3^ or the presence of an AIDS-defining event, with pooled data from COHERE and EuroSIDA cohorts indicating that approximately 53.8% of PLWH are classified as LP (44). In Eastern Europe, the pooled prevalence of late presentation has been reported to be approximately 48.4%, with substantial variation between countries. Consistent with these reports, a high proportion of late presenters was observed in our study population as well. Late presentation and advanced immunodeficiency have been associated with an increased burden of comorbidities, including MS, in several studies of PLWH receiving ART (45). However, in our cohort, neither the degree of immunodeficiency at the presentation nor CD4+ T-cell strata were significantly associated with the development of MS, and no genotype-specific differences were observed with respect to the *HIF1α* rs11549465 polymorphism. These findings are in line with data from multiple large cohort and case–control studies demonstrating that advanced immunodeficiency at the time of HIV diagnosis is not an independent risk factor for MS in PLWH on ART (37, 46). Possible explanation for this observation is that PLWH with advanced HIV infection often experience substantial weight loss, particularly involving visceral and abdominal adipose tissue, which may limit the fulfilment of MS criteria – especially WC – thereby creating a misleading appearance of reduced metabolic risk. In addition, ethnic differences and the use of varying diagnostic criteria for MS across studies may further contribute to heterogenous findings in this field (46, 47).

To date, a limited number of studies have examined the association between *HIF1α* gene polymorphisms and the development of MS. To the best of our knowledge, the study of Zafar et al. is the only one demonstrating a protective association between the *HIF1α* Pro582Ser CT genotype and MS in the general population (48). However, comparable studies in PLWH are lacking, despite their well-documented increased risk for developing MS. In this context, our study is the first to demonstrate a protective association of the *HIF-1α* rs11549465 T allele and CT genotype with MS in Caucasian individuals living with HIV and receiving ART. The *HIF1α* Pro582Ser polymorphism affects the HIF-1α protein in its C-terminal transactivation domain (C-TAD), leading to alterations in protein stability, transcriptional activity, and regulatory control. Experimental evidence indicates that the proline-to-serine substitution at position 582 reduces the susceptibility of HIF-1α to hyperglycemia-induced inhibition under hypoxic conditions, thereby preserving its stability and functional activity when it would be suppressed otherwise (49). Functional reporter assays demonstrate enhanced activation of canonical HIF-1 target genes, including VEGF, EPO, and GLUT1, likely mediated by increased recruitment of p300/CBP co-activators by the Pro582Ser variant during hypoxia (50). As a result, downstream hypoxia-responsive pathways such as angiogenesis, glycolysis, and glucose transport may be more effectively activated, potentially conferring metabolic and vascular protection in ischaemic and diabetic context (9). The apparent protective effect observed predominantly in heterozygous CT carriers of the Pro582Ser (rs11549465) polymorphism, rather than in TT homozygotes, is consistent with previous reports and may reflect several factors: the low minor allele frequency of the TT allele limits statistical power for analyses involving TT genotypes, while the heterozygous state may provide a functional equilibrium that optimizes HIF1α activity (51). This pattern is compatible with the principle of heterozygote advantage (overdominance), whereby heterozygous individuals may demonstrate superior functional outcomes compared to homozygous state, a phenomenon well described in other biological contexts, such as the sickle-cell trait (52).

This study demonstrated that PLWH who developed MS while receiving ART exhibited significantly higher SBP and DBP values. Hypertension is highly prevalent among PLWH, with meta-analysed indicating a higher burden in individuals on ART compared to the general population. Its prevalence is exceeding 50% among PLWH older than 50 years (53). HIV-associated hypertension is linked to increased cardiovascular morbidity and mortality, and it is thought to result from a combination of long-term inflammation, persistent immune activation, ART-related metabolic alterations, and dysregulation of the renin-angiotensin-aldosterone system (RAAS) and the sympathetic nervous system (54, 55). Additional contributing factors include microbial translocation, endothelial dysfunction, and increased salt sensitivity, all of which appear to be more pronounced in PLWH (56). Current evidence suggests that the *HIF-1α* rs11549465 (C1772T) polymorphism may influence cardiovascular risk phenotypes. The T allele, particularly in the heterozygous CT state, has been associated with protection against severe hypertensive disorders, such as pre-eclampsia and HELLP syndrome (57), as well as with broader susceptibility to cardiovascular and metabolic complications in Caucasian populations (58). In line with these observations, our findings indicate a protective association of the CT+TT genotype with the occurrence of hypertension among PLWH who developed MS, with a similar trend observed in the control group. These reports support a potential modulatory role of *HIF1α* genetic variation in blood pressure regulation in the context of HIV-related metabolic disease. However, as this study was exploratory and included multiple secondary comparisons, the results should be considered preliminary and require validation in larger populations.

Our results confirmed significantly higher fasting glycemia levels in participants with MS compared to controls, as expected for a syndrome-defining component. Previous studies have reported a protective effect of the T allele of *HIF-1α* rs11549465 against type 1 and type 2 DM in Caucasian and Japanese populations (50, 59, 60), as well as against diabetic complications such as retinopathy and nephropathy. On the other hand, Pichu et al. (61) observed that Indian patients with DM and diabetic foot ulcers had a higher frequency of the T allele, suggesting a maladaptive hypoxic response in chronic ischemic conditions. However, the CT+TT genotype was not significantly associated with fasting glycemia among PLWH with MS in our cohort. Dyslipidemia is highly prevalent among PLWH and shares multiple pathophysiological mechanisms with MS, including the effect of HIV proteins, chronic inflammation, immune activation, microbial translocation, and ART (62). These processes contribute to elevated TG and LDL-C levels and reduced high-density lipoprotein (HDL) levels (63, 64), thereby increasing cardiovascular risk, as reflected in the protective value of lipid ratios such as TC/HDL-C (65). Although HIF-1α is a known to influence lipid metabolism by promoting triglyceride synthesis and modifying fatty acid utilization under hypoxic conditions (66, 67), no genetic studies have examined *HIF-1α* rs11549465 in relation to dyslipidemia. Consistent with this gap in the literature, our study did not identify a significant association between *HIF-1α* rs11549465 genotypes and lipid abnormalities in either cases or controls.

In our cohort, PLWH who developed MS while receiving ART demonstrated significantly higher WC and BMI values compared to the control group. Although central obesity was defined using WC in line with the MS criteria, BMI was also examined as a broader indicator of overall overweight/adiposity, which may provide additional context when interpreting metabolic risk in treated PLWH. Although substantial experimental evidence supports the involvement of HIF-1α’s role in adipose tissue physiology and obesity-related inflammation, data linking the *HIF-1α* Pro582Ser variant to anthropometric obesity remain scarce. To date, no published studies have demonstrated a direct association between this polymorphism and BMI or WC in human population. A candidate-gene analysis in a Malay cohort revealed significance only within a combined genotype model of HIF1α and NFκB1 variants, without isolating the effect of rs11549465 (68). In line with these observations, we did not detect a significant association between the CT genotype and obesity-related traits in either PLWH with MS or controls.

Our results showed that the duration of ART was associated with the occurrence of MS in PLWH, while among ART classes, PI-based regimens were most commonly linked to the development of MS. Importantly, the inverse association between the CT genotype and MS was observed irrespective of ART regimen. While genotype × ART interactions were not statistically significant, the modest size of some genotype strata may have reduced the precision of these estimates, and the results should be viewed in that context. There is strong evidence that the duration of HIV infection and ART exposure is associated with a higher prevalence of MS among PLWH, as confirmed by large global cohort studies (69–71) and supported by our findings. Most cohorts suggest that cumulative HIV infection duration and/or prolonged ART exposure – particularly with PIs and older NRTIs – increase the risk of MS; however, in some large samples, the association is attenuated after adjustment, for factors such as age and BMI, implying the mediating influence of obesity, aging, and specific drug-related effects (41, 72), that are also observed in our final regression models. Our results further support the notion that ART strategies in our cohort evolved over time, with a clear shift from PI-dominated initial therapy to INSTI-based regimens at index visit, after a median ART duration of 10 years. Given that PIs predominated in early treatment phases, their contribution to the development of the MS should be considered, particularly through mechanisms such as impaired GLUT4-mediated glucose transport (73). In addition, thymidine analogues have been linked to mitochondrial toxicity and lipoatrophy (74). Notably, nearly 57% of participants in our cohort have been exposed to abacavir as part of combination ART at some point. Multiple large cohort studies have consistently demonstrated the association between abacavir exposure and cardiovascular events (75). Although the underlying mechanisms are not fully understood, proposed pathways include activation of inflammatory biomarkers such as high-sensitivity C-reactive protein (hsCRP) and interleukin-6 (IL-6), endothelial dysfunction promoting atherosclerosis, and increased platelet aggregation and reactivity (76).

The findings of this study suggest that the association between the CT genotype of *HIF-1α* Pro582Ser and lower odds of MS was more evident in PLWH with shorter HIV and ART duration, while this pattern was less apparent in those with longer exposure. Having in mind the study design and sample size, causal inferences cannot be firmly established. However, these findings support a potential time-dependent effect, whereby the initial metabolic benefits associated with enhanced HIF-1α activity – particularly on glucose and lipid regulation – may gradually be outweighed by persistent HIV-related inflammation, cumulative ART-associated metabolic toxicity, and aging-related metabolic stress. As a result, the observed protective association appeared stronger in the early course of HIV infection and less evident with prolonged exposure. Future research involving larger cohorts and longitudinal design are needed to clarify this relationship and to determine whether the attenuation of the association reflects ART-related metabolic effects, HIV-associated immune dysregulation, or their synergistic interaction.

The association between *HIF-1α* rs11549465 and MS remained statistically significant after adjusting for demographic and HIV-related factors in primary regression model, suggesting that the finding was not explained by age, duration of HIV infection and ART group. Although the cross-sectional study design does not allow conclusions about causality, the consistency of the association after multivariable adjustment supports the hypothesis of a biologically relevant genetic contribution. In the secondary multivariable model incorporating the core NCEP ATP III components, individuals with the *HIF1α* Pro582Ser T allele demonstrated significantly lower odds of MS, with an approximate 83% odds reduction, irrespective of clinical covariates. Age, SBP, and BMI were independently associated with MS, while abnormal glycemia and elevated TG levels showed the strongest associations, as expected for syndrome-defining components. The duration of HIV infection lost its significance after adjustment, indicating that its effect may be mediated through cumulative cardiometabolic factors rather than acting independently. The broad CIs for rs11549465 and for glycemia/TG reflect limited precision, likely due to the small sample size and the low frequency of TT genotypes. Replication of these findings in larger cohorts with longitudinal study design and formal testing of the interaction between genotype and HIV infection/ART duration are warranted.

This study has several limitations. The relatively small sample size could have limited statistical power; however, the results represent pioneering research in this area, as comparable studies in PLWH are scarce. The small number of *HIF-1α* rs11549465 TT carriers limited meaningful evaluation of a recessive model. Similarly, excluding participants receiving metabolic therapy from continuous analyses resulted in smaller analytic subsets, which may have reduced statistical sensitivity. Accordingly, the findings should be interpreted primarily as hypothesis-generating rather than confirmatory. Furthermore, other potentially functional *HIF-1α* polymorphisms were not assessed, and the potential influence of unmeasured factors, such as environmental influences or the use of other medications cannot be excluded. Finally, we did not assess messenger RNA (mRNA) or HIF-1α levels, which would have provided further mechanistic insight.

## 5 Conclusion

Our results show that the T allele and CT/CT+TT genotypes of the *HIF-1α* rs11549465 polymorphism are associated with lower odds of prevalent MS in PLWH receiving ART. The protective association appeared more evident among individuals with shorter duration of HIV infection and ART exposure, suggesting a possible time-related pattern which requires further research. Although the cross-sectional design does not allow inference about causality, these results support the hypothesis that hypoxia-related genetic variation may contribute to interindividual differences in metabolic susceptibility among PLWH and may have relevance for future risk stratification and long-term metabolic management.

## Data Availability

The datasets presented in this study can be found in online repositories. The names of the repository/repositories and accession number(s) can be found in the article/Supplementary material.

## Footnotes

1
http://www.hapmap.org/

2
http://www.ncbi.nlm.nih.gov/projects/SNP/

## Glossary

AIDS: Acquired immunodeficiency syndrome
ART: Antiretroviral therapy
ATH: Adipose tissue hypoxia
BH: Body height
BMI: Body mass index
BP: Blood pressure
BW: Body weight
CD4+: CD4-positive T helper cells
CDC: Centers for Disease Control and Prevention
95% CI: 95% confidence interval
C-TAD: C-terminal transactivation domain
DBP: Diastolic blood pressure
DM: Diabetes mellitus
dsDNA: Double-stranded deoxyribonucleic acid
EDTA: Ethylenediaminetetraacetic acid
FMNKg: Faculty of Medical Sciences, University of Kragujevac
HDL-C: High-density lipoprotein cholesterol
HIF: Hypoxia-Inducible Factor
HIF-1α: Hypoxia-Inducible Factor 1α
HIV RNA: Human Immunodeficiency Virus Ribonucleic Acid
hsCRP: High-sensitivity C-reactive protein
HTA: Hypertension
IDF: International Diabetes Foundation
IQR: Interquartile range
IR: Insulin resistance
LD: Lipodystrophy
LDL-C: Low-density lipoprotein cholesterol
LP: Late presenters
Md: Median
MS: Metabolic syndrome
NCEP ATP III: National Cholesterol Education Program Adult Treatment Panel III
non-LP: non-Late presenters
NRTI: Nucleoside reverse transcriptase inhibitor
NNRTI: Non-nucleoside reverse transcriptase inhibitor
OR: Odds ratio
PCR: Polymerase Chain Reaction
PI: Protease inhibitor
PLWH: People Living with HIV
RAAS: Renin-Angiotensin-Aldosterone System
RNA: Ribonucleic acid
ROS: Reactive Oxygen Species
RT-PCR: Reverse Transcription-Polymerase Chain Reaction
S.E.: The standard error
SBP: Systolic blood pressure
SNPs: Single nucleotide polymorphisms
TC: Total cholesterol
TG: Triglycerides
UKCKg: University Clinical Center Kragujevac
UKCS: University Clinical Center of Serbia
Wald *χ*^2^: Wald test statistics
WC: Waist circumference
