# Supplementary material for: Protective association of the HIF-1α rs11549465 polymorphism with metabolic syndrome in people living with HIV on antiretroviral therapy
Source: Front Med (Lausanne). 2026 Mar 31;13:1802450. doi: 10.3389/fmed.2026.1802450 (PMC13076327; doi:10.3389/fmed.2026.1802450)
Supplement: Supplementary file 1 [file Data_Sheet_1.PDF]

Supplementary table 1. Genotype × ART class cross-tabulated cell counts stratified by MS status. Genotype was coded using the dominant genetic model (CT+TT vs CC). ART class reflects the regimen at the index visit (NNRTI, PI and INSTI). Absolute counts are shown in order to illustrate genotype distribution across ART groups and the size of each subgroup used in the interaction analyses. Abbreviations: NNRTI - Non-nucleoside reverse transcriptase inhibitors; PI - protease inhibitors; INSTI - Integrase strand transfer inhibitors;

**Study group**			**rs11549465 genotype**		**Total**
			**CC**	**CT+TT**	
**MS**	ART group	NNRTI-based	4	1	5
		PI-based	5	4	9
		INSTI-based	23	10	33
	Total		32	15	47
**Control**	ART group	NNRTI-based	7	10	17
		PI-based	5	3	8
		INSTI-based	22	27	49
	Total		34	40	74
**Total**	ART group	NNRTI-based	11	11	22
		PI-based	10	7	17
		INSTI-based	45	37	82
	**Total**		66	55	121
